# Nasal irrigation efficiently attenuates SARS-CoV-2 Omicron infection, transmission and lung injury in the Syrian hamster model

**DOI:** 10.1016/j.isci.2022.105475

**Published:** 2022-11-02

**Authors:** Lunzhi Yuan, Huachen Zhu, Ming Zhou, Jian Ma, Xuan Liu, Kun Wu, Jianghui Ye, Huan Yu, Peiwen Chen, Rirong Chen, Jia Wang, Yali Zhang, Shengxiang Ge, Quan Yuan, Tong Cheng, Yi Guan, Ningshao Xia

**Affiliations:** 1 State Key Laboratory of Molecular Vaccinology and Molecular Diagnostics, National Institute of Diagnostics and Vaccine Development in Infectious Diseases, School of Life Sciences, School of Public Health, Xiamen University, Xiamen, Fujian, China; 2 State Key Laboratory of Emerging Infectious Diseases, School of Public Health, Li Ka Shing Faculty of Medicine, The University of Hong Kong, Hong Kong SAR, China; 3 Guangdong-Hong Kong Joint Laboratory of Emerging Infectious Diseases/Joint Laboratory for International Collaboration in Virology and Emerging Infectious Diseases, Joint Institute of Virology (STU/HKU), Shantou University, Shantou, Guangdong, China; 4 EKIH (Gewuzhikang) Pathogen Research Institute, Futian District, Shenzhen, Guangdong, China

**Keywords:** Immunology, Virology, Immune response

## Abstract

Recently, a new variant lineage of SARS-CoV-2, namely Omicron, became the dominant global circulating strain. The multiple antigenic mutations of Omicron largely decrease the efficiency of current vaccines and neutralizing antibodies, which highlights the need for more potent and reachable medical countermeasures. Here, we hypothesize that direct viral clearance by nasal irrigation might be a convenient and alternative option, and perform proof-of-concept experiments in the Syrian hamster model. Interestingly, Omicron shows a different dynamic in the changes of viral RNA, viral titers, and proinflammatory cytokines in nasal rinsing samples when compared with the prototype. Meanwhile, the levels of viral load and proinflammatory cytokines in nasal rinsing samples can indicate the severity of lung injury. Of note, daily nasal irrigation efficiently attenuates inflammation and lung injury in Omicron-infected hamsters by decreasing the viral loads in the respiratory tract organs. Moreover, daily nasal irrigation effectively suppresses viral transmission by close contact.

## Highlights

• Daily nasal irrigation suppresses SARS-CoV-2 replication and close contact transmission
• Daily nasal irrigation significantly decreases cytokine storm and diffuse lung injury caused by SARS-CoV-2
• Viral load and cytokines in nasal rinsing samples indicate the severity of lung injury

## Graphical abstract

**Figure:**
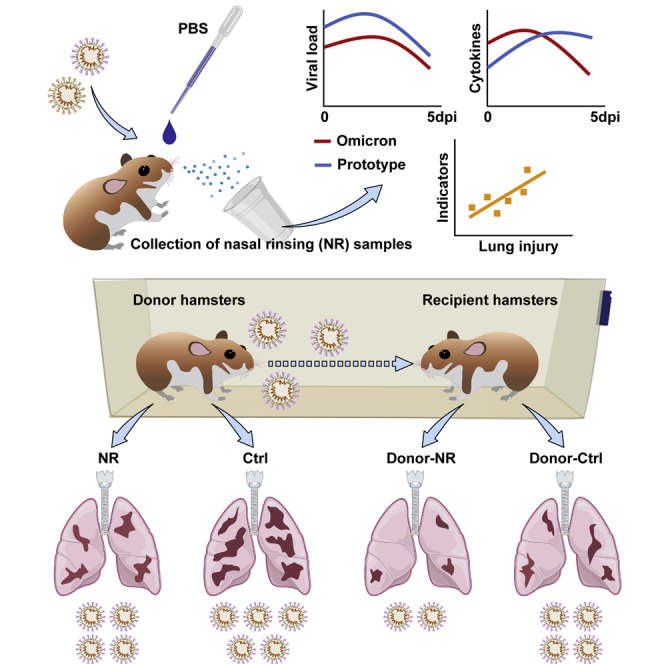

## Introduction

Severe acute respiratory syndrome coronavirus 2 (SARS-CoV-2) infects more than 500 million people worldwide and causes coronavirus diseases 2019 (COVID-19) with mild or severe clinical symptoms including cough, fever, and pneumonia. SARS-CoV-2 is highly infectious in respiratory tract tissues, resulting in robust viral shedding and airborne transmission.^1^^,^^2^ It also evades cells through the tight binding between its surface spike glycoprotein and the host receptor, angiotensin-converting enzyme 2 (ACE2).^3^ Therefore, the vast majority of vaccines and neutralizing antibodies are designed to block SARS-CoV-2 infection by targeting the spike protein.^4^^,^^5^^,^^6^^,^^7^^,^^8^ However, their protective and therapeutic efficiency can be largely hampered by the high genomic mutation rate in SARS-CoV-2 variants.^9^ The most widely spreading emerging SARS-CoV-2 variants include Alpha, Beta, Gamma, Delta and Omicron.^10^ In contrast to the prototype virus, the variants have gained variations in their infectivity, transmissibility, pathogenicity, and antigenicity by different mutations. For instance, the critical substitutions in Omicron have led to immune escape from the convalescent and vaccinated serum, causing breakthrough infections among the fully vaccinated populations even with a booster.^11^^,^^12^^,^^13^^,^^14^ Interestingly, Omicron showed increased transmissibility but decreased pathogenicity when compared with the prototype virus and the other variant lineages.^15^ It is reported that Omicron infection is associated with high viral RNA levels in nasopharyngeal swabs and exhaled aerosols.^16^^,^^17^ However, viral RNA, viral titer and pathological changes in pulmonary tissues were less in Omicron-infected animal models than in those infected with prototype SARS-CoV-2 and other variants.^18^^,^^19^

Despite the decreasing efficiency of vaccines, practical treatment after SARS-CoV-2 infection is still lacking. Although antiviral drugs have been approved, their benefits are impeded by several drawbacks. For instance, the vast majority of the neutralizing antibodies available to previous SARS-CoV-2 variants are invalid to Omicron.^20^ Intravenous and oral administration of antiviral compounds such as Remdesivir, Molnupiravir and Paxlovid have good preventive effects, but less therapeutic efficiency to suppress viral replication and lung injury.^21^^,^^22^^,^^23^^,^^24^ Moreover, these drugs were too expensive to afford by the low-income groups and people in the developing countries. Therefore, a more affordable, reachable, convenient, and efficient option is earnestly demanded to combat the COVID-19 pandemic. Recently, several case reports showed that nasal irrigation can relieve the clinical symptoms of COVID-19 and suppress viral load.^25^^,^^26^^,^^27^ Clinical researchers in south China found that nasal irrigation might accelerate viral clearance and recovery of COVID-19 patients with infections of the Omicron variant. However, randomized and parallel controlled experiment is still lacking, and the detailed therapeutic mechanism is unclear. Therefore, we set up to test the therapeutic efficiency of nasal irrigation against COVID-19 in the well-accepted Syrian hamster model.^28^^,^^29^ Firstly, we detected the dynamic changes of viral load and proinflammatory cytokines in nasal rinsing (NR) samples and investigated whether nasal irrigation was effective in treating COVID-19 caused by SARS-CoV-2 prototype and Omicron strains. Secondly, we analyzed the correlation between lung injury and the levels of viral RNA, titer, and proinflammatory cytokines in NR samples at the endpoint of the animal experiment. Thirdly, we examined whether nasal irrigation was able to suppress close contact transmission of SARS-CoV-2. Overall, we performed a proof-of-concept study to evaluate the practical value of nasal irrigation and interpreted its therapeutic mechanism in the Syrian hamster model, which might feasibly serve as a non-pharmaceutical option for therapy of COVID-19 patients with SARS-CoV-2 Omicron infection.

## Results

### Dynamics of viral load and proinflammatory cytokines in nasal rinsing samples

**Figure 1:**
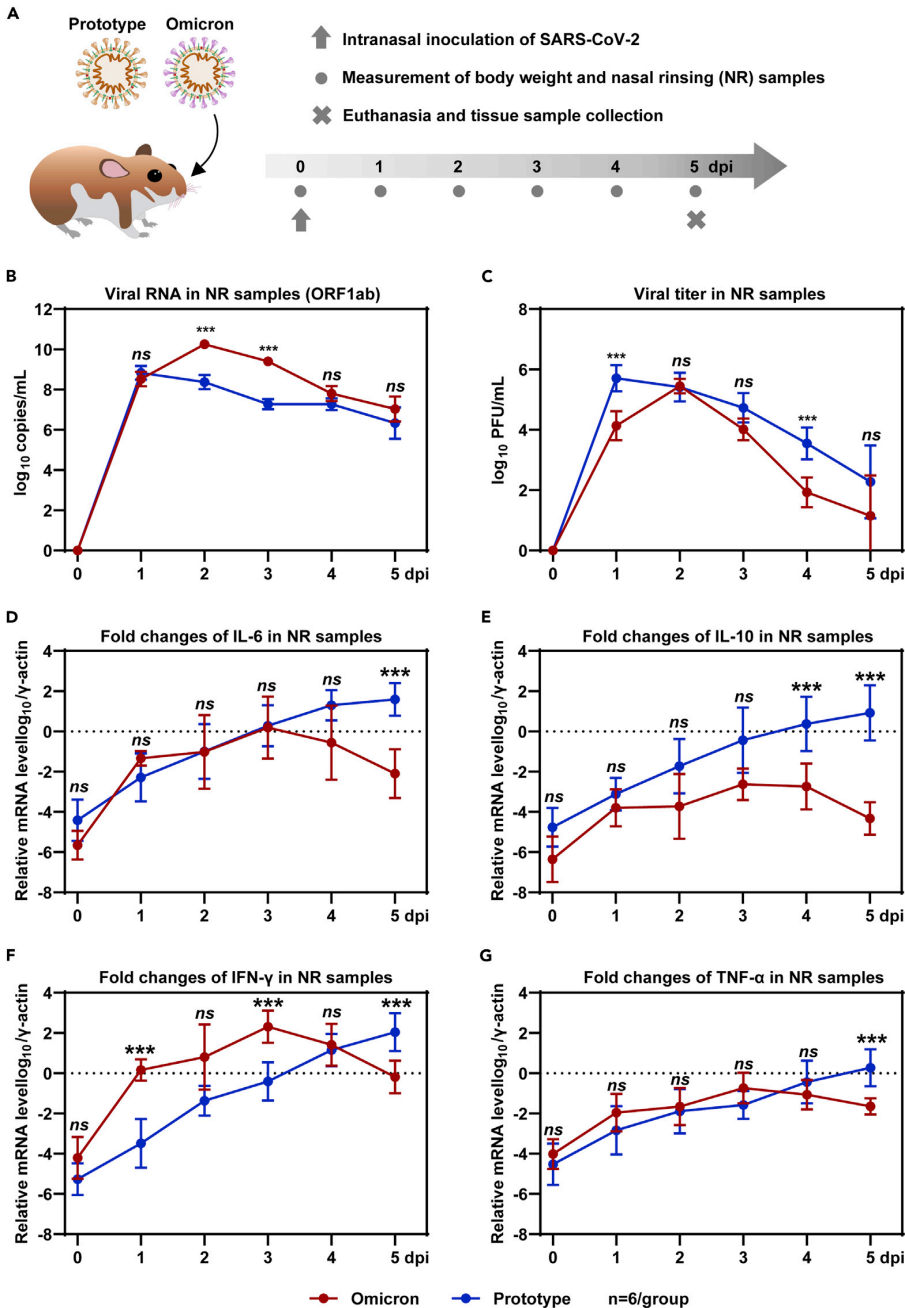
Viral load and mRNA levels of proinflammatory cytokines in NR samples (A) Schematic diagram of SARS-CoV-2 infection and animal experiments. Hamsters were intranasally inoculated with 1 × 10^5^ PFU of prototype SARS-CoV-2 and Omicron BA.1 variant, respectively. Bodyweight changes were daily measured and NR samples were daily collected from 0 to 5 dpi. Animals were euthanized at 5 dpi for virological and pathological analysis. (B) Viral RNA and (C) viral titer in the NR samples were measured by RT-PCR and plaque assays (n = 6/group). Fold changes for mRNA levels of proinflammatory cytokines including those of (D) IL-6, (E) IL-10, (F) IFN-γ and (G) TNF-α in the NR samples of the hamsters were measured by RT-PCR (n = 6/group). The mRNA levels of proinflammatory cytokines were standardized to the house-keeping gene γ-actin. Significance is calculated using two-way ANOVA.

In order to mimic patients of COVID-19 in animal models, male Syrian hamsters were intranasally inoculated with 1×10^5^ plaque-forming units (PFU) of SARS-CoV-2 Omicron BA.1 variant or the prototype virus as previously described.^28^^,^^29^ Physical and health examinations were undertaken by recording their survival rate and body weight changes. Nasal rinsing with 1 mL of phosphate buffer solution (PBS) was taken from 0 to 5 days post-inoculation (dpi) to measure the levels of viral load and proinflammatory cytokines in the turbinate mucosa interface (Figure 1A). All of the SARS-CoV-2 infected hamsters survived at 5 dpi. We analyzed the viral replication in NR samples by real-time reverse transcription polymerase chain reaction (RT-PCR) that amplified SARS-CoV-2 open reading frame 1ab (ORF1ab) for the detection of viral RNA load. Viral titers of these samples were measured by half tissue culture infective dose (TCID_50_). Generally, NR samples collected from Omicron BA.1-infected hamsters showed a higher viral RNA, but a lower viral titer than those collected from prototype SARS-CoV-2-infected hamsters from 0 to 5 dpi. The Omicron BA.1-infected hamsters showed 7.04 ± 0.62 log_10_ copies/mL of viral RNA (Figure 1B) and 1.15 ± 1.34 log_10_ TCID_50_/mL of viral titer (Figure 1C) in NR samples at 5 dpi, while the prototype-infected hamsters showed 6.33 ± 0.78 log_10_ copies/mL of viral RNA (Figure 1B) and 2.28 ± 1.21 log_10_ TCID_50_/mL of viral titer (Figure 1C).

The severity of COVID-19 is closely related to abnormal immune response and inflammation. Previous studies have focused on the uncontrolled release of proinflammatory cytokines in solid organ tissues and blood, but the dynamic changes of proinflammatory cytokines in turbinate mucosa interface were rarely known. We thus measured the mRNA levels of several proinflammatory cytokines in the NR samples by RT-PCR. Compared to the hamsters infected with prototype SARS-CoV-2, the Omicron BA.1-infected ones showed significantly lower mRNA levels of interleukin 6 (IL-6), IL-10, interferon γ (IFN-γ) and tumor necrosis factor-alpha (TNF-α) in NR samples at 5 dpi (Figures 1D-1G), indicating a milder inflammation.

### Daily nasal irrigation decreased the disease severity in SARS-CoV-2-infected hamsters

**Figure 2:**
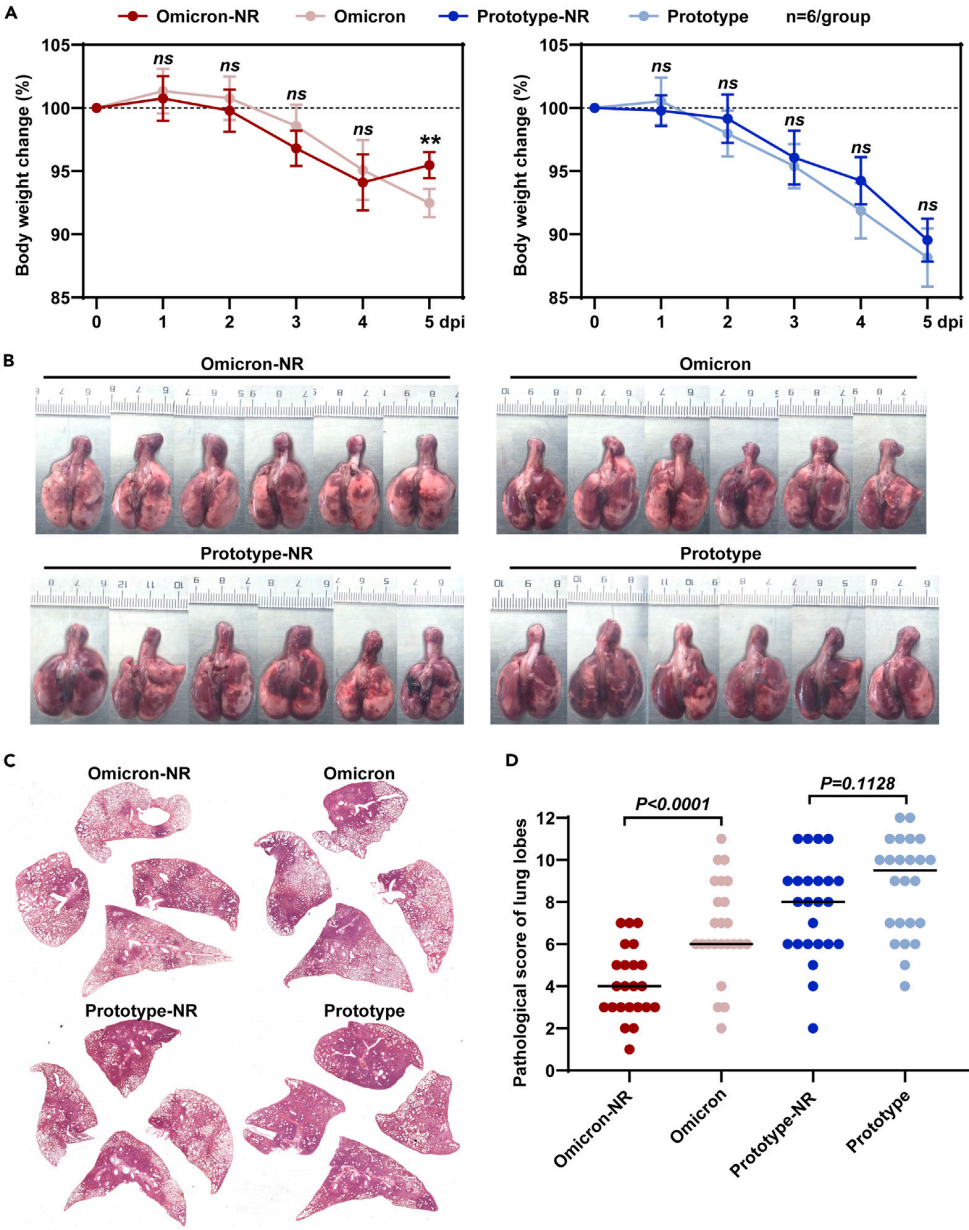
Physiological changes of SARS-CoV-2 infected hamsters with or without daily nasal irrigation (A) Body weight changes of the SARS-CoV-2 prototype and Omicron BA.1 variant infected hamsters with or without daily nasal irrigation (n = 6/group). Significance is calculated using two-way ANOVA. (B) Gross images of lung tissues. (C) Representative H&E staining for lung lobe sections collected from the SARS-CoV-2 infected hamsters at 5 dpi. H&E staining for all the rest of the hamsters were shown in Figure S3. (D) Comprehensive pathological scores for lung sections were determined based on the severity and percentage of injured areas of each lung lobe. For each group, more than 20 lung lobes were collected from six individual hamsters and were scored (Table S1). Significance is calculated using one-way ANOVA.

To examine whether nasal irrigation is able to treat COVID-19, we performed physiological, virological, and pathological analyses of the SARS-CoV-2 infected hamsters with and without nasal irrigation. In contrast to the Omicron BA.1-infected hamsters without nasal irrigation, the ones with daily nasal irrigation showed a rescue of body weight loss at 5 dpi (Figure 2A), which indicated a recovery of health state. However, nasal irrigation did not rescue body weight loss in the hamsters infected with prototype SARS-CoV-2 (Figure 2A). To evaluate the severity of lung pathology, all the hamsters were euthanized at 5 dpi. First, we anatomized the animals and isolated their lungs for gross observation. As expected, Omicron BA.1 variant has a decreased pathogenicity than the SARS-CoV-2 prototype strain. Severe lung lesions including consolidation, multifocal, and diffuse hyperemia were seen in the prototype-infected hamsters with or without nasal irrigation (Figure 2B). In the Omicron-infected hamsters, nasal irrigation significantly relieved the severity of lung lesions (Figure 2B). To have a comprehensive analysis of lung pathological changes, lung lobes were fixed in formalin for hematoxylin and eosin (H&E) staining. The lung lobes of SARS-CoV-2 prototype infected hamsters with or without nasal irrigation showed typical features of severe pneumonia including lung lobe consolidation and alveolar destruction, diffusive inflammation, protein-rich fluid exudate, hyaline membrane formation, and severe pulmonary hemorrhage (Figures 2C and S1). The Omicron-infected hamsters showed features of middle to moderate pneumonia. Furthermore, nasal irrigation further reduced the severity of lung pathogenesis in the hamsters infected with Omicron BA.1 variant (Figures 2C and S1). In addition, the severity of lung pathology was quantified by comprehensive pathological scoring based on alveolar septum thickening and consolidation, hemorrhage, exudation, pulmonary edema and mucous, recruitment and infiltration of inflammatory cells among all the hamster lung lobes (Figure 2D and Table S1). The Omicron BA.1 variant and SARS-CoV-2 prototype infected hamsters with and without nasal irrigation showed average comprehensive pathological scores of 4.13 ± 1.69, 6.71 ± 2.28, 7.67 ± 2.33 and 8.75 ± 2.31, respectively (Figure 2D).

**Figure 3:**
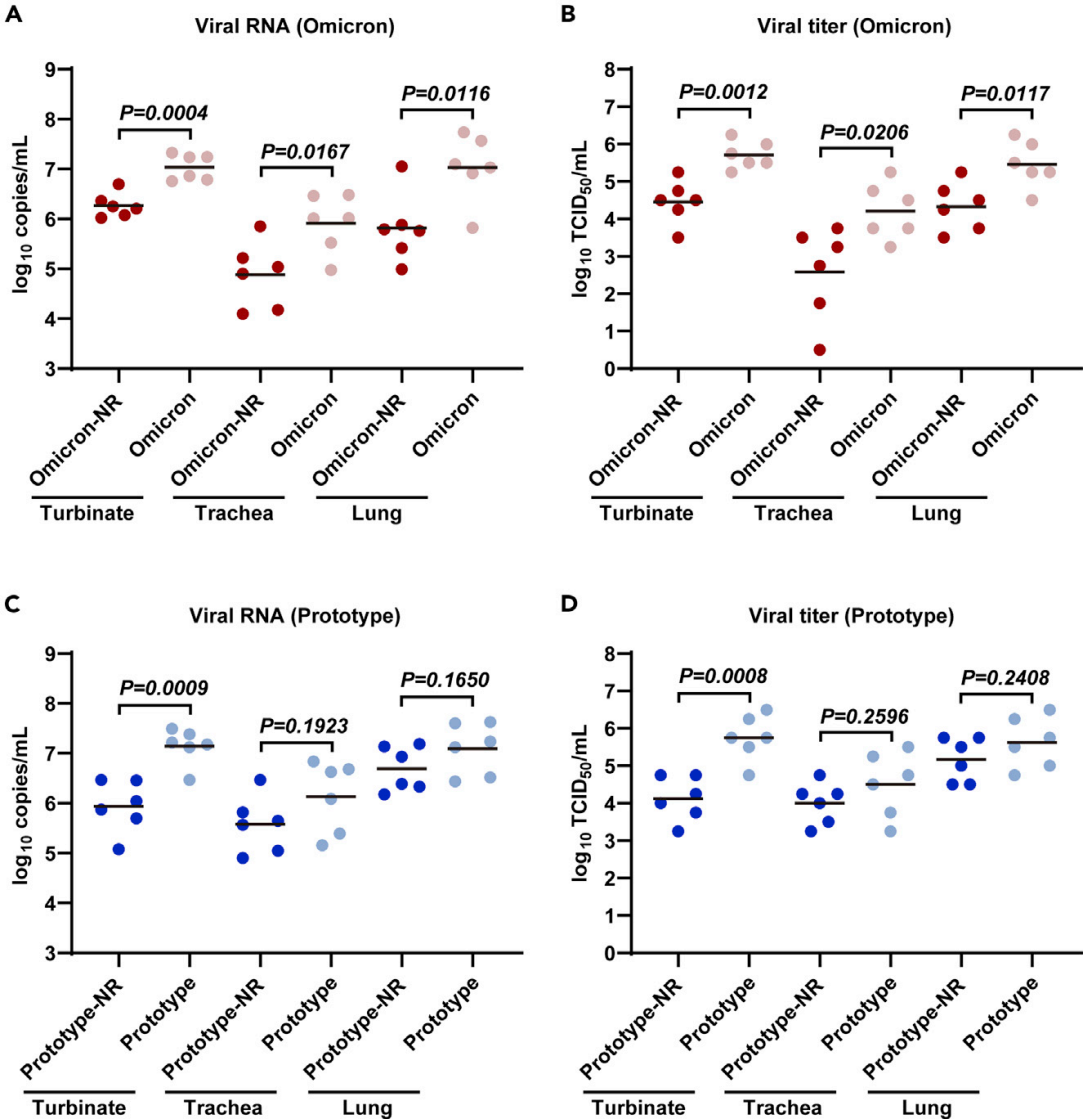
Viral load in main respiratory organs of SARS-CoV-2 infected hamsters with or without daily nasal irrigation Viral RNA levels from turbinate, trachea, and lung tissues of SARS-CoV-2 (A) Omicron BA.1 variant and (C) prototype infected hamsters with or without nasal irrigation were measured by RT-PCR (n = 6/group), using primers to amplify the SARS-CoV-2 ORF1ab gene. (B and D) Viral titers of indicated tissue samples were measured by half tissue culture infective dose (TCID_50_) titration method (n = 6/group). Significance is calculated using one-way ANOVA.

**Figure 4:**
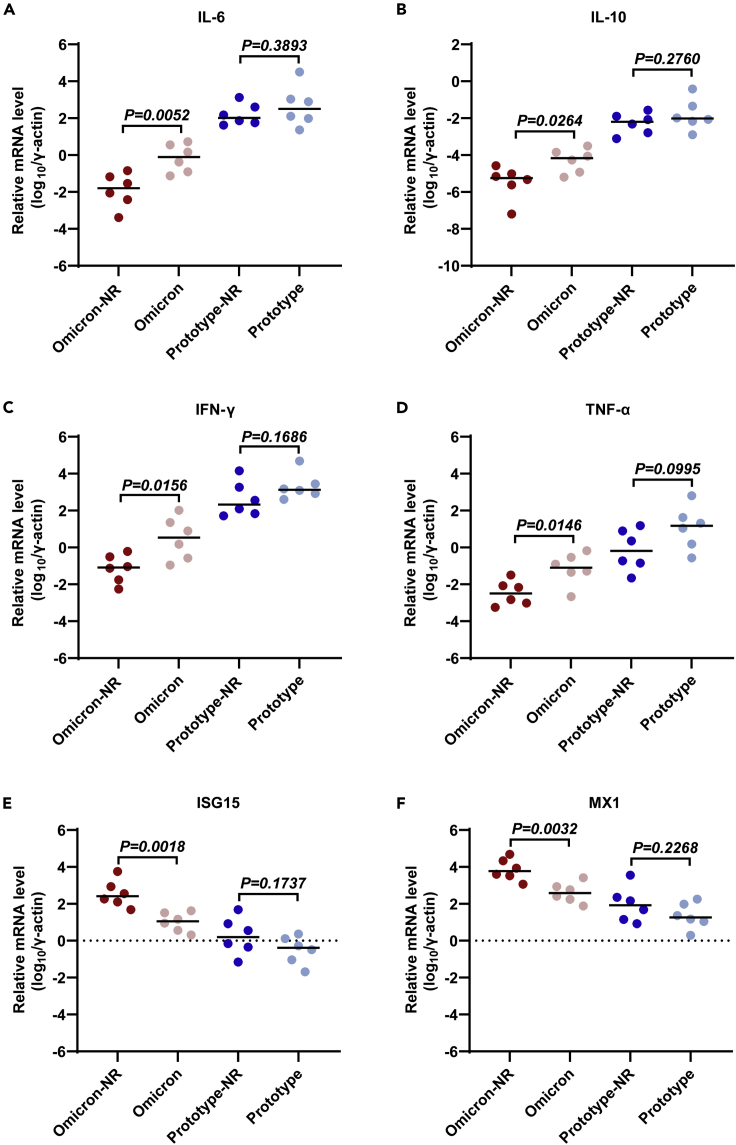
Measurement for mRNA levels of proinflammatory cytokines in lung tissues Fold changes for mRNA levels of proinflammatory cytokines including those of (A) IL-6, (B) IL-10, (C) IFN-γ, (D) TNF-α, (E) ISG15 and (F) MX1 in the lung tissues of hamsters sacrificed at 5 dpi were measured by RT-PCR (n = 6/group).

Then, we asked whether daily nasal irrigation could affect viral replication in respiratory tract organs. To this end, we analyzed viral replication in respiratory tract organs including turbinate, trachea, and lung by RT-PCR that amplified the SARS-CoV-2 ORF1ab gene for the detection of viral RNA load in the homogenized tissues collected at 5 dpi. In the Omicron BA.1-infected hamsters, daily nasal irrigation significantly reduced both the viral RNA and viral titers in respiratory tract organs. The Omicron-infected hamsters with daily nasal irrigation showed 6.27 ± 0.24, 4.88 ± 0.65, 5.82 ± 0.69 log_10_ copies/mL of viral RNA in turbinate, trachea, and lung, respectively (Figure 3A), and 4.46 ± 0.58, 2.59 ± 1.24, 4.33 ± 0.65 log_10_ TCID_50_/mL of viral titer (Figure 3B). Those without treatment showed 7.04 ± 0.26, 5.91 ± 0.58, 7.02 ± 0.67 log_10_ copies/mL of viral RNA (Figure 3A) and 5.71 ± 0.37, 4.21 ± 0.75, 5.46 ± 0.62 log_10_ TCID_50_/mL of viral titer (Figure 3B) comparatively. Notwithstanding, the viral load in respiratory tract organs of SARS-CoV-2 prototype infected hamsters with daily nasal irrigation was only slightly but not significantly reduced (Figures 3C and 3D). In addition, the results of immunohistochemistry staining for SARS-CoV-2 nucleocapsid protein (NP) in lung sections suggested that daily nasal irrigation can suppress the positive rate of SARS-CoV-2 NP in the lungs of Omicron BA.1-infected hamsters, but not in the ones infected with the prototype virus (Figure S5). Next, we measured the relative mRNA levels of proinflammatory cytokines in lung tissues. IL-6, IL-10, IFN-γ, and TNF-α in Omicron BA.1-infected hamsters were significantly suppressed by nasal rinsing (Figures 4A-4D). However, the decrease of proinflammatory cytokines was slight in SARS-CoV-2 prototype infected hamsters with nasal rinsing (Figures 4A-4D). Meanwhile, nasal irrigation is able to increase the mRNA levels of two critical interferon stimulated gene (ISG) including ISG15 and myxovirus resistance protein 1 (MX1) in the lung tissues of hamsters infected with both the prototype and Omicron BA.1 (Figures 4E and 4F), suggesting an enhanced type one interferon response.

Afterward, we tested whether the time interval of nasal irrigation can affect the therapeutic effect in a parallel experiment. In the Omicron BA.1-infected hamsters, the animals with twice nasal irrigation at 1 and 3 dpi showed an attenuated therapeutic effect than those with daily nasal irrigation from 1 to 5 dpi (Figures S5 and S6 and Table S2). First, the hamsters with twice nasal irrigation showed no rescue of body weight loss at 5 dpi. Second, the relief of lung pathological changes and the suppression of viral RNA and titer in respiratory tract organs have been weakened. These findings suggested that reducing the time interval of nasal irrigation is able to improve its therapeutic effect. Taken together, daily nasal irrigation was able to decrease the disease severity in SARS-CoV-2 Omicron BA.1 variant infected hamsters by suppressing viral load in respiratory tract organs and proinflammatory cytokine levels in lungs.

### Daily nasal irrigation was able to reduce transmission of SARS-CoV-2 Omicron BA.1 variant in hamsters by close contact

**Figure 5:**
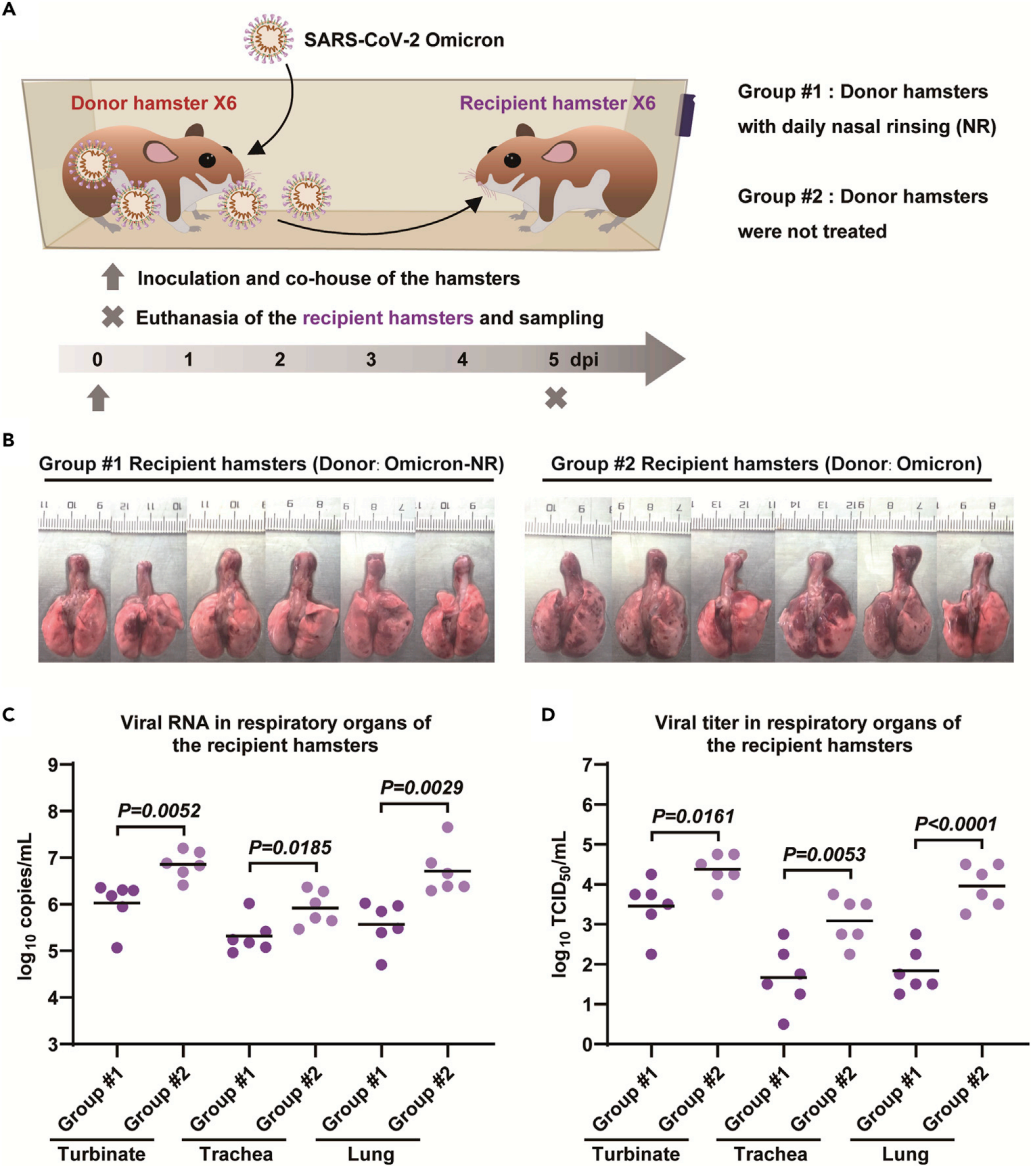
Attenuation of the close contact transmission of SARS-CoV-2 by daily nasal irrigation (A) Schematic diagram of close contact infection of SARS-CoV-2 Omicron BA.1 variant. In brief, six donor hamsters were intranasally inoculated with 1 × 10^5^ PFU of SARS-CoV-2 Omicron BA.1 variant and then co-housed with six recipient hamsters for five days. In the experiment group (group #1), NR samples of the donor hamsters were daily collected from 0 to 5 dpi. In the control group (group #2), the donor hamsters were not treated. Afterward, the recipient hamsters were euthanized at 5 dpi for virological and pathological analysis. (B) Gross images of lung tissues. Viral RNA levels from (C) turbinate, trachea, and lung tissues of the recipient hamsters were measured by RT-PCR (n = 6/group), using primers to amplify the SARS-CoV-2 ORF1ab gene. (D) Viral titers of indicated tissue samples were measured by TCID_50_ titration method (n = 6/group). Significance is calculated using one-way ANOVA.

As the viral load in respiratory tract organs were suppressed in the Omicron BA.1-infected hamsters by daily nasal irrigation, we thus tested whether virus transmission of these animals was reduced in a close contact model of Syrian hamster infected with SARS-CoV-2. In brief, six donor hamsters were intranasally inoculated with 1 × 10^5^ PFU of SARS-CoV-2 Omicron BA.1 variant and then co-housed with six recipient hamsters for five days. In the nasal rinsing group (group #1), nasal irrigation of the donor hamsters was daily performed from 0 to 5 dpi. In the control group (group #2), the donor hamsters were not treated. Afterward, the recipient hamsters were euthanized at 5 dpi for virological and pathological analysis (Figure 5A). Gross images of lung tissues of the recipient hamsters showed that the infection of Omicron BA.1 variant via close contact induced a milder lung pathology than those infected by intranasal inoculation (Figure 5B). Daily nasal irrigation further reduced the severity of lung pathology (Figure 5B). Next, we analyzed viral replication in respiratory tract organs including turbinate, trachea, and lung of the recipient hamsters by RT-PCR that amplified SARS-CoV-2 ORF1ab gene for the detection of viral RNA load in the homogenized tissues collected at 5 dpi. In contrast to the recipient hamsters of group #2, the recipient hamsters of group #1 showed 5-10 folder lower viral RNA levels in these respiratory organs (Figure 5C). The results of viral titers in these organs were consistent with those of the viral RNA levels (Figure 5D). Overall, these data suggested that daily nasal irrigation was able to reduce transmission of the SARS-CoV-2 Omicron BA.1 variant in hamsters by close contact.

## Discussion

Although the SARS-CoV-2 Omicron variant has a relatively lower pathogenicity than the prototype virus and the other variants of concerns, its high transmissibility has led to a robust increase of infection cases.^15^ Although the vast majority are non-hospitalized patients, they still exist as infectious sources in the community.^30^^,^^31^^,^^32^^,^^33^ Moreover, some of them might further develop into critical COVID-19,^34^^,^^35^^,^^36^ suggesting that observation, diagnosis, early intervention, and treatment are still necessary. Therefore, rational management of these patients remains a big challenge to reduce the transmission and infection of the SARS-CoV-2 Omicron variant.

Most of the current medical countermeasures are based on biological approaches to defend against SARS-CoV-2 infection and treat COVID-19. In this study, we attempt to use nasal irrigation, a convenient physical method, to achieve direct viral clearance and relief of symptoms. Of note, our animal experiments in Syrian hamsters demonstrated that daily nasal irrigation is an affordable, reachable, convenient operation and efficient medical countermeasure to combat the Omicron variant. In contrast to the hamsters infected with the SARS-CoV-2 prototype strain, the Omicron BA.1 variant infected hamsters showed higher viral RNA load but lower viral titer in the NR samples collected from 1 to 5 dpi. These data suggested a decrease in viral shedding but not the viral RNA replication of Omicron BA.1 variant in upper respiratory tract tissues, especially from 2 to 5 dpi. Combined with the sharply decreasing mRNA levels of proinflammatory cytokines in the NR samples since 3 dpi, the low viral titer indicated that the viral replication of the Omicron BA.1 variant has been rapidly controlled by innate immunity. Indeed, the Omicron BA.1-infected hamsters with daily nasal irrigation showed a significant loss of both viral RNA and viral titer in turbinate, trachea, and lung at 5 dpi, respectively. Therefore, we speculated that the operation of nasal irrigation can directly clear the infectious virus particles in nasal mucosa, mucus and aerosol, subsequentially decrease the viral shedding from the upper to the lower respiratory tracts, and finally result in a systematical suppression of viral load in turbinate, trachea and lung. Apart from the inhibition of viral replication and removal of thick mucus with debris in the nasal passages, daily nasal flushing effectively reduced the levels of proinflammatory cytokines secreted from lung and respiratory tissues, which have been considered the main drivers of COVID-19.^37^^,^^38^ As expected, the Omicron BA.1-caused symptoms of body weight loss and lung injury were significantly relieved by daily nasal irrigation.

Remarkably, the viral RNA and proinflammatory cytokine levels in NR samples can precisely predict the severity of lung injury in the Omicron BA.1 variant infected hamsters. These findings revealed the internal connection between the upper and lower respiratory tracts and provided several useful indicators as diagnostic or prognostic markers to guide disease stratification and evaluation of severity. In contrast to the Omicron BA.1 variant infected hamsters, nasal irrigation treatment was less efficient in those infected with the SARS-CoV-2 prototype virus. This is mainly because the infectivity and pathogenicity of the Omicron BA.1 variant in lungs is largely attenuated, with the virus mostly replicating in the upper respiratory tract.^18^^,^^19^ Meanwhile, the spike glycoprotein of the Omicron variant is likely to induce a relatively weaker host immune response,^39^ which indicates an adequate control of viral replication with lower inflammatory tissue injury. Therefore, daily nasal irrigation, as a mild and convenient procedure to clear congested sinuses, has been sufficient to relieve the Omicron infections and associated symptoms. However, the SARS-CoV-2 prototype and early variants usually trigger unregulated host immune response with delayed viral clearance, uncontrolled release of proinflammatory cytokines, and robust tissue injury.^40^ Indeed, the variant-specific pathogenicity features of SARS-CoV-2 prototype and Omicron BA.1 variant were faithfully represented in the hamster model of this study. In summary, our study suggested that direct viral clearance in upper respiratory tract might be helpful to suppress viral load and inflammatory injury in lower respiratory tract, especially for the SARS-CoV-2 variants with relatively low pathogenicity, such as Omicron.

Another important application of nasal irrigation is to reduce the high transmissibility of the Omicron BA.1 variant. Although daily nasal irrigation cannot completely block viral transmission in the close contact infection of Omicron BA.1 variant from the donors to the recipient hamsters, approximately 10- to 100-fold decrease of viral RNA and viral titer were detected in turbinate, trachea, and lung of the recipient hamsters at 5 dpi, respectively. These results demonstrated that the infectious virus particles in exhaled aerosols of the donor hamsters were largely reduced by nasal irrigation, which might represent an accelerating negative conversion of nasopharyngeal swabs and suppressed viral shedding in clinical practice. As we performed daily nasal irrigation in this study, multiple nasal irrigation per day might achieve an enhanced viral clearance and transmission blockade. Besides, the use of medical apparatus such as sprayer and atomizer might further improve therapeutic efficiency, operation conveniency, and comfort level. For clinical translation, the amount and component of rinsing buffer, collection, and detection of the infectious samples need to be well considered and set as standard operating procedures. Moreover, additional anti-inflammatory agents in the rinsing buffer might be an effective supplement to enhance the therapeutic effect of nasal irrigation.^41^

Altogether, the overarching data in this study demonstrated that nasal irrigation is a reachable and convenient medical countermeasure to treat mild and asymptomatic patients with SARS-CoV-2 Omicron variant infection in hospital or in-house quarantine, especially in countries and regions without adequate public health and medical sources. In addition to current countermeasures such as mass vaccination, isolating cases, and maintaining social distance, nasal irrigation provides a new approach to help us defend against SARS-CoV-2. We believe our findings will be helpful to control the widespread transmission of the SARS-CoV-2 Omicron variant, the other newly emerging SARS-CoV-2 variants, and the analogous respiratory tract pathogens in the foreseeable future.

### Limitations of the study

We did not test the therapeutic efficiency of nasal irrigation in COVID-19 animal models with larger body sizes, such as ferrets, mink, and non-human primates.

## STAR★Methods

### Key resources table

**Table:** 

REAGENT or RESOURCE	SOURCE	IDENTIFIER
**Bacterial and virus strains**		

SARS-CoV-2 prototype	This study	AP-8
SARS-CoV-2 Omicron BA.1	This study	AP-309
SARS-CoV-2 NP antibody	This study	15A7-1
Vero cells	ATCC	#CCL-81

**Critical commercial assays**		

Dulbecco’s Modified Eagle Medium (DMEM)	GIBCO	#11995
Fetal bovine serum (FBS)		#10270106
Phosphate buffer saline (PBS)		#10010031
TPCK-trypsin	SIGMA-ALDRICH	#T1426
DMSO		#D5879
Penicillin-Streptomycin	Invitrogen	#15140-122
MgCl_2_	Thermo Fisher	#AM9530G
Isoflurane	RWD Life Science	#R510-22
Fast-King Strand cDNA Synthesis Kit	TIANGEN	#FP313
QIAamp Viral RNA Mini kit	Qiagen	#52906
RNeasy Mini Kit		#74106
SARS-CoV-2 RT-PCR Kit	Wantai	N/A
Testosterone	Shanghai meilian	#ml050081
RNAlater™ Stabilization Solution	Invitrogen	#AM7021
Hematoxylin	Maxim Biotechnology	#CTS-1096
Eosin		#CTS-4094
Slide		#SLI-20010312

### Resource availability

#### Lead contact

Further information and requests for resources and reagents should be directed to and will be fulfilled by the lead contact, Ningshao Xia (nsxia@xmu.edu.cn).

#### Materials availability

All unique/stable reagents generated in this study are available from the lead contact with a completed materials transfer agreement.

### Experimental model and subject details

#### Experimental animal and biosafety

Six- to eight-week-old male hamsters (LVG strain) were used in this study. The Syrian Hamster was raised in the specific pathogen free animal feeding facilities. All the animal experiments were approved by the Medical Ethics Committee (SUMC2021-112). All experiments with infectious SARS-CoV-2 were performed in the biosafety level 3 (BSL-3) and animal biosafety level 3 (ABSL-3) facilities. Our staff wore powered air-purifying respirators that filtered the air, and disposable coveralls when they cultured the virus and handled animals that were in isolators. The researchers were disinfected before they left the room and then showered on exiting the facility. All facilities, procedures, training records, safety drills, and inventory records were subject to periodic inspections and ongoing oversight by the institutional biosafety officers who consult frequently with the facility managers.

#### Virus stock

The SARS-CoV-2 prototype AP-8 (EPI_ISL_1655937), and Omicron BA.1 variant AP-309 (share an identical sequence with EPI_ISL_8182026) were passaged on Vero cells (#CCL-81, ATCC). Viral stocks were prepared in Vero cells with DMEM containing 2% FBS, 5ug/mL TPCK-trypsin, 1% Penicillin-Streptomycin and 30mmol/L MgCl_2_. Viruses were harvested and stored in ultra-low temperature freezer. The titers were determined by means of plaque assay in Vero cells.

### Method details

#### Virus inoculation and sample collection

We collected the initial nasal rinsing samples at day 0, and then inoculated the hamsters with SARS-CoV-2. Six- to eight-week-old male hamsters were anesthetized by isoflurane (#R510-22, RWD Life Science) and nasally inoculated with indicated doses of SARS-CoV-2 diluted in 200μL of PBS (#10010031, GIBCO). In the next five days, we collected the nasal rinsing samples at the same time point. Body weight of these hamsters were measured by an electronic balance. Hamsters were euthanized at the indicated time point for detection of viral load in respiratory tract organs and pathological examination in lung lobes.

#### Administration of nasal irrigation

Hamsters were well fixed in 45-degree up in the biological safety cabinet, and 1mL of PBS (with 2% Penicillin-Streptomycin) was slowly instilled into the two nostrils by using a plastic dropper. Nasal rinsing samples were then collected into a plastic sampling cup and transferred into a sampling tube for storage or further detection.

#### Detection of viral RNA and viral titer

For the solid organ samples, we collect 1mg turbinate, 0.1 mg trachea and 0.1 mg lung in 1mL PBS for homogenate and detection of viral RNA and viral titer. Viral RNA was extracted by using a QIAamp Viral RNA Mini kit (#52906, Qiagen) according to the manufacturer’s instructions. The RT-PCR was conducted by using the SLAN-96S Real-Time System (Hongshi, Shanghai, China) with a SARS-CoV-2 RT-PCR Kit from Wantai (Beijing, China). Relative Viral RNA of SARS-CoV-2 ORF1ab gene was determined using primer pairs and probes provided in the kit. Viral RNA copies were expressed on a log_10_ scale after normalized to the standard curve obtained by using ten-fold dilutions of a SARS-CoV-2 stock. The titer of viral stock, nasal rinsing samples and homogenized tissues were measured by plaque assay and half tissue culture infective dose (TCID_50_) titration method in Vero cells seeded in 6-well and 96-well plates, respectively.

#### Detection of cytokine mRNA

The lung tissues were cleaved into small pieces and soaked in RNAlater (#AM7021, Invitrogen). Total RNAs in lysed lung tissues and nasal rinsing samples were extracted with RNeasy Mini kit (#74106, Qiagen) and reverse-transcribed into cDNAs with Fast-King Strand cDNA Synthesis Kit (#FP313, TIANGEN, Beijing). Diluted cDNAs (1:10) were quantified using SYBR Green I-based real-time PCR using the LightCycler® 480 instrument (Roche) per manufacturer’s instructions. Threshold cycle (Ct) of each gene was normalized to the internal reference gene (hamster γ-actin) and comparative Ct (2-ΔΔCt) method was utilized to calculate changes in chemokine and cytokine gene expression profile. The gene-specific primers (5′ to 3′) used for RT-PCR were listed in Table S3.

#### Histopathological studies

For pathological analysis, lung tissues were fixed in formalin for more than 72 hours, dehydrated and then embedded in paraffin wax. The wax block of lung tissues was cut into 4μm sections for pathological staining and analysis. H&E staining was employed for analysis of general lung pathogenic lesions including pulmonary edema, consolidation and inflammation. The standards for pathological score of lung tissues in this study are derived from our previous study in hamster model. Comprehensive pathological scoring of lung sections was performed according to the degree of lung lesions including alveolar septum hyperplasia, consolidation and impairment of alveolar structure, fluid exudation, mucus suppository, thrombus, inflammation recruitment and infiltration of immune cells in each individual lung lobe. For each hamster, three or four lung lobes were employed for evaluation of comprehensive pathological score. In brief, H&E staining result of each lung lobe was analyzed for its severity of pathological change. The pathological score included: a) Alveolar septum thickening and consolidation; b) Hemorrhage, exudation, pulmonary edema and mucous; c) Recruitment and infiltration of inflammatory immune cells. For each issue, scores were related to the severity: 0 indicated no pathological change was observed, 1 indicated moderate pathological change, 2 indicated mild pathological change, 3 indicated severe pathological change and 4 indicated very severe pathological change. In conclusion, scores of such three issues were added as the comprehensive pathological score of a lung lobe, and the average comprehensive pathological score of the lobes indicated the severity of lung pathology in an evaluated hamster. Immunohistochemistry staining for SARS-CoV-2 nucleocapsid protein (NP) was employed for analysis of viral antigen expression and distribution in lung tissues. A mouse anti SARS-CoV-2 N protein specific antibody (15A7-1, house-keeping) was used as the first antibody of immunohistochemistry staining. The pathological reagents include immunohistochemistry kit (#KIT-9730), Hematoxylin (#CTS-1096) and Eosin (#CTS-4094) were purchased from Maxim Biotechnology (Fuzhou, China). The images of whole lung lobes were screened by a high-throughput screening microscope system (EVOS M7000, Invitrogen of Thermo Fisher Scientific).

### Quantification and statistical analysis

#### Animal and sample size justification

Sample sizes maximized considering limits in BSL-3 working capacity, numbers of animals that can be handled under ABSL-3 conditions and availability of well-trained staffs.

#### Statistical analysis

Student’s unpaired two-tailed t-test and one-way ANOVA were performed using GraphPad Prism 8.0 (GraphPad Software). Data are presented as the means ± SD. Two-sided p-values <0.01 were considered significant: ∗p < 0.01, ∗∗p < 0.001, ∗∗∗p < 0.0001, NS indicates no significance.

## Data Availability

This study did not generate any unique datasets or code.
