# Supplementary material for: Nasal irrigation efficiently attenuates SARS-CoV-2 Omicron infection, transmission and lung injury in the Syrian hamster model
Source: iScience. 2022 Nov 2;25(12):105475. doi: 10.1016/j.isci.2022.105475 (PMC9625844; doi:10.1016/j.isci.2022.105475)
Supplement: Document S1. Figures S1–S6 and Tables S1–S3 [file mmc1.pdf]

iScience, Volume *25*

Supplemental information

Nasal irrigation efficiently attenuates SARS-CoV-2 Omicron infection, transmission and lung injury in the Syrian hamster model

Lunzhi Yuan, Huachen Zhu, Ming Zhou, Jian Ma, Xuan Liu, Kun Wu, Jianghui Ye, Huan Yu, Peiwen Chen, Rirong Chen, Jia Wang, Yali Zhang, Shengxiang Ge, Quan Yuan, Tong Cheng, Yi Guan, and Ningshao Xia

Supplementary materials

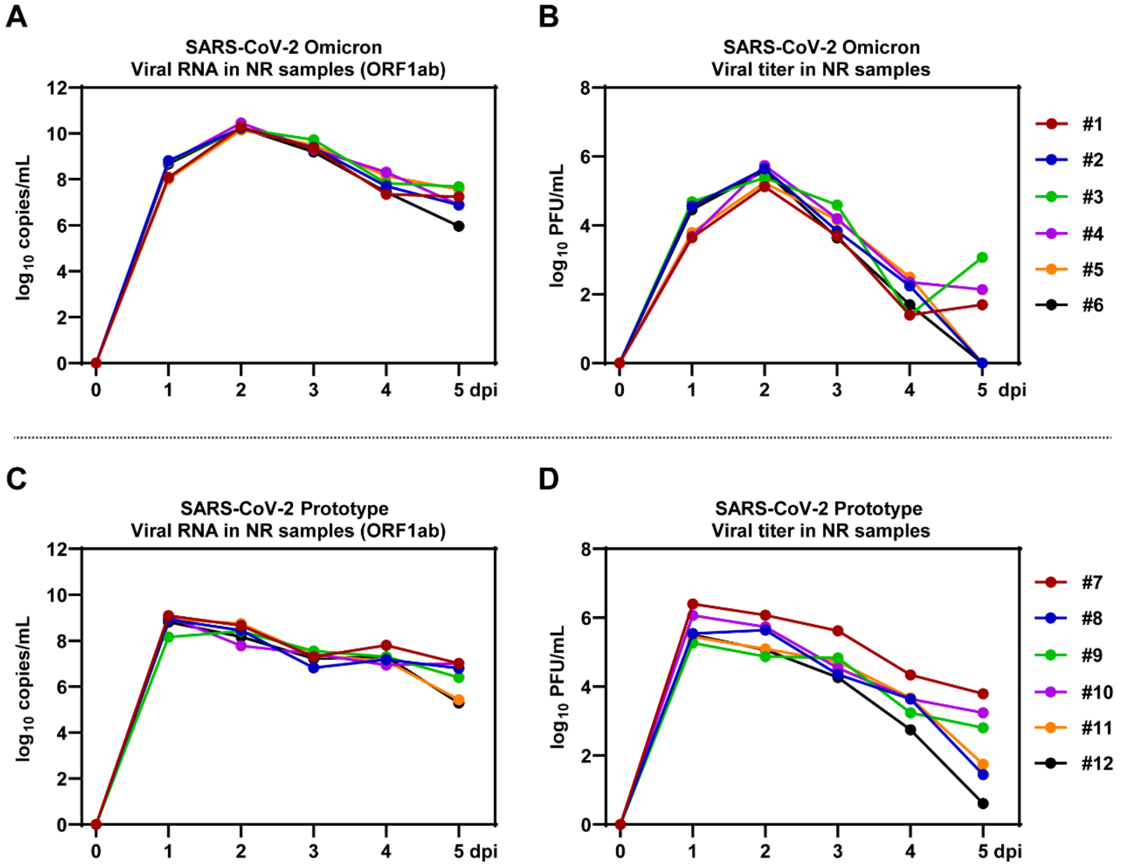

Supplementary Figure 1. Detection of viral load in the daily nasal rinsing (NR) samples of individual hamster infected with SARS-CoV-2, related to Figure 1. Viral RNA and viral titer in the NR samples of hamsters infected with **(A, B)** Omicron BA.1 variant and **(C, D)** SARS-CoV-2 prototype strain were measured by RT-PCR and $TCID_{50}$ titration method.

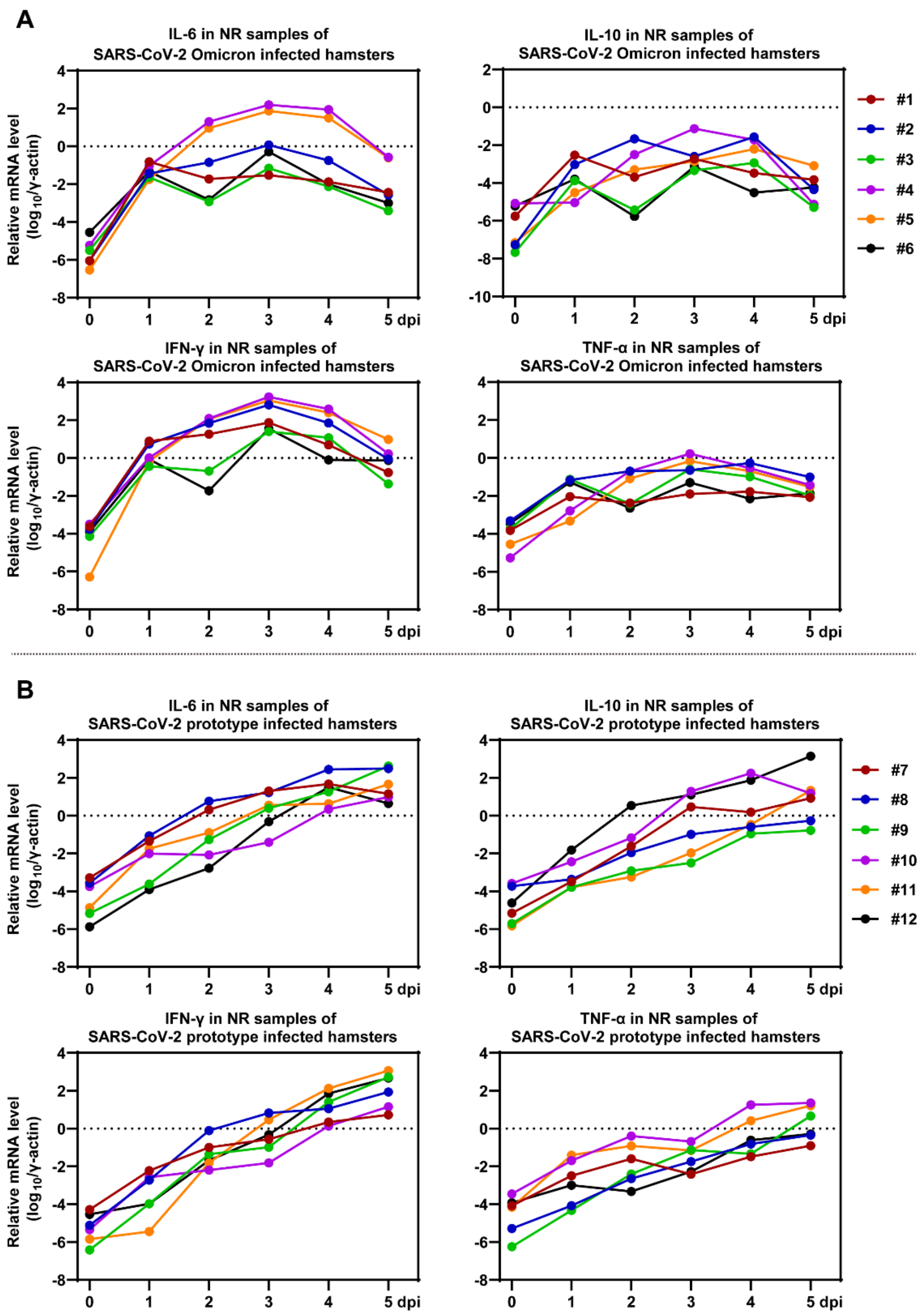

Supplementary Figure 2. Detection of proinflammatory cytokines in the daily NR samples of individual hamster infected with SARS-CoV-2, related to Figure 1. Fold changes for mRNA levels of proinflammatory cytokines including those of IL-6, IL-10, IFN-γ and TNF-α in the NR samples of hamsters infected with **(A)** Omicron BA.1 variant and **(B)** SARS-CoV-2 prototype strain were measured by RT-PCR (n=6/group). The mRNA levels of proinflammatory cytokines were standardized to the house-keeping gene γ-actin.

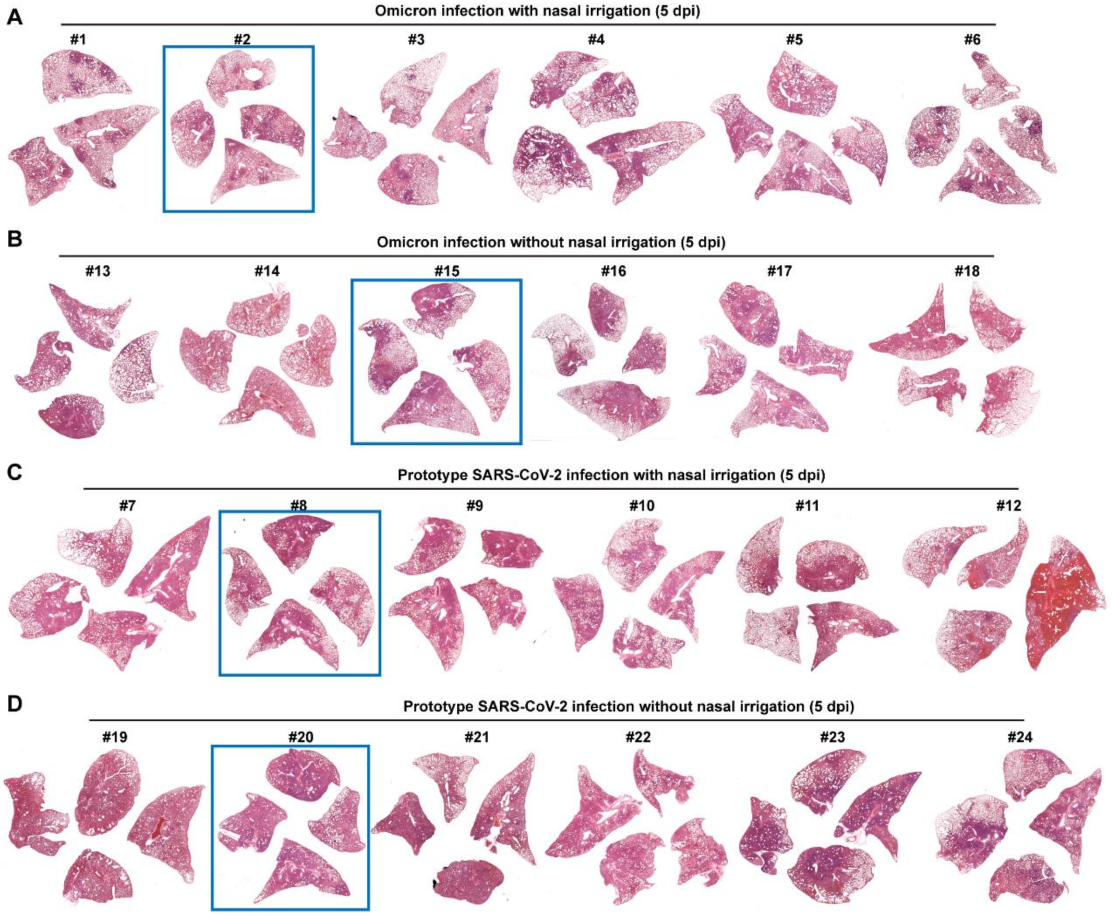

Supplementary Figure 3. H&E staining of lung lobes collected from SARS-CoV-2-infected hamsters with or without nasal irrigation, related to Figure 2. All of the hamsters were sacrificed at 5 dpi. For each hamster, three or four lung lobes were fixed in formalin for pathological analysis. H&E staining for lung lobe sections from **(A)** the Omicron BA.1 variant infected hamsters with daily nasal rinsing, **(B)** the Omicron BA.1 variant infected hamsters without daily nasal rinsing, **(C)** the SARS-CoV-2 prototype infected hamsters with daily nasal rinsing, and **(D)** the SARS-CoV-2 prototype infected hamsters without daily nasal rinsing. The representative images in blue boxes were shown in Figure 2.

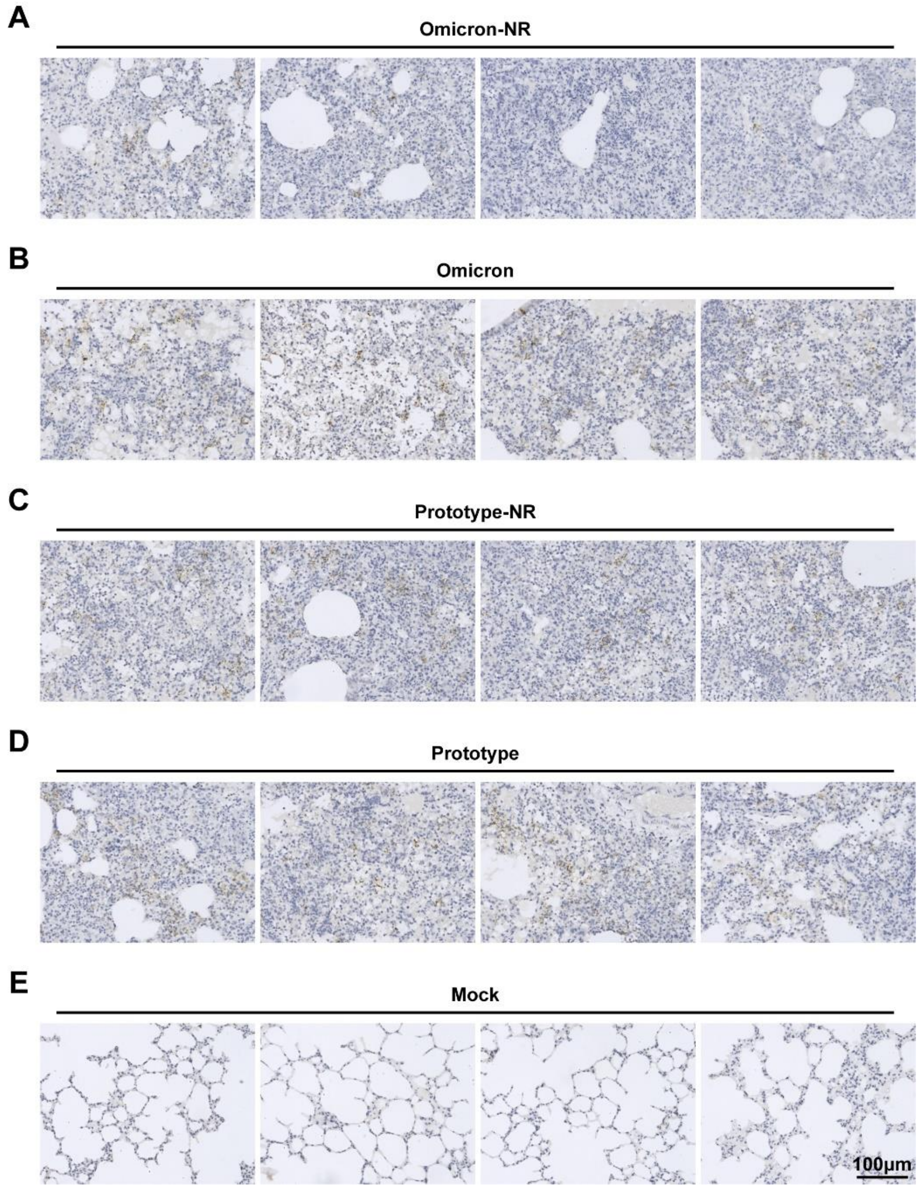

Supplementary Figure 4. Immunohistochemistry staining for SARS-CoV-2 nucleocapsid protein in the hamster lung lobes, related to Figure 2. All of the hamsters were sacrificed at 5 dpi. For each hamster, three or four lung lobes were fixed in formalin for pathological analysis. H&E staining for lung lobe sections from **(A)** the Omicron BA.1 variant infected hamsters with daily nasal rinsing, **(B)** the Omicron BA.1 variant infected hamsters without daily nasal rinsing, **(C)** the SARS-CoV-2 prototype infected hamsters with daily nasal rinsing, **(D)** the SARS-CoV-2 prototype infected hamsters without daily nasal rinsing and **(E)** the mock hamsters without SARS-CoV-2 infection (bar=100μm).

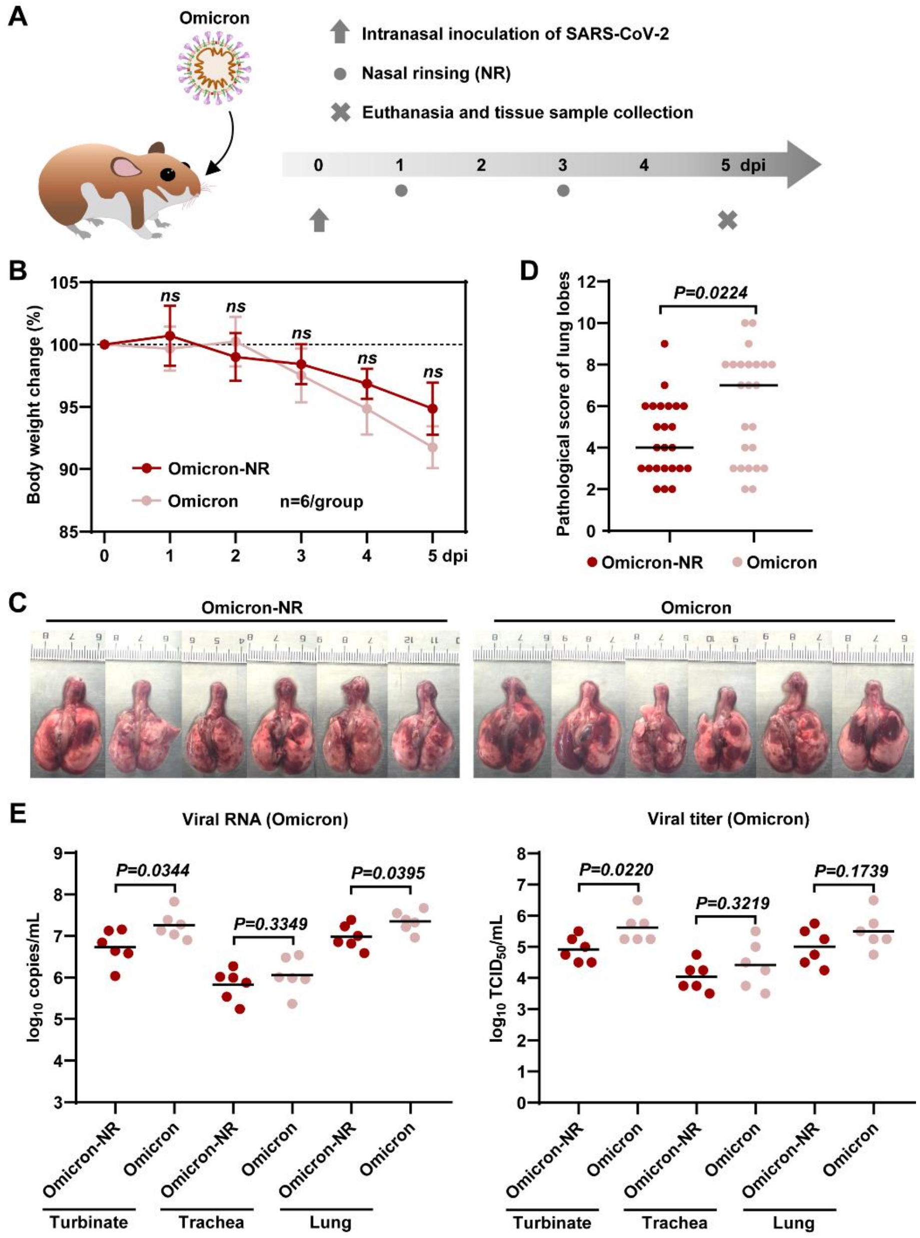

Supplementary Figure 5. Physiological changes and virological of Omicron BA.1-infected hamsters with or without nasal irrigation, related to Figure 2. (A) Scheme for virus inoculation, twice nasal irrigation at 1 and 3 dpi and sample collection. **(B)** Body weight changes from 0 to 5 dpi (n=6/group). Significance is calculated using two-way ANOVA. **(C)** Gross images of lung tissues collected at 5 dpi. **(D)** Comprehensive pathological scores for lung sections were determined based on the severity and percentage of injured areas of each lung lobe. For each group, more than 20 lung lobes were collected from six individual hamsters and were scored (Figure S6 and Table. S2). Significance is calculated using one-way ANOVA. **(E)** Viral RNA levels from turbinate, trachea and lung tissues were measured by RT-PCR (n=6/group),

using primers to amplify the SARS-CoV-2 ORF1ab gene. Viral titers of indicated tissue samples were measured by $TCID_{50}$ titration method (n=6/group). Significance is calculated using one-way ANOVA.

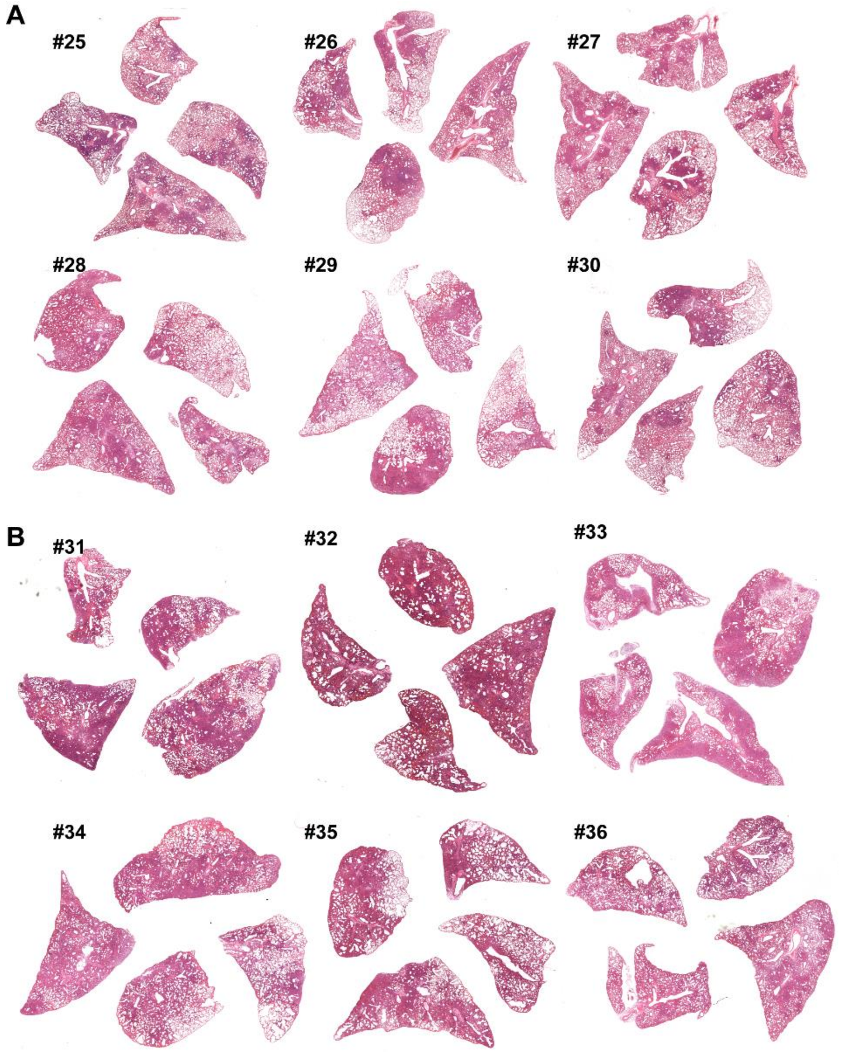

Supplementary Figure 6. H&E staining of lung lobes collected from Omicron BA.1-infected hamsters with or without twice nasal irrigation, related to Figure 2. All of the hamsters were sacrificed at 5 dpi. For each hamster, three or four lung lobes were fixed in formalin for pathological analysis. H&E staining for lung lobe sections from **(A)** the Omicron BA.1 variant infected hamsters with twice nasal rinsing and **(B)** the Omicron BA.1 variant infected hamsters without nasal rinsing.

Table S1. Comprehensive pathological scores of the SARS-CoV-2 Omicron variant and prototype infected hamsters sacrificed at 5 dpi, related to Figure 2.

Group	No.	Pathological lesions			Comprehensive pathological score	Average
		Alveolar septum hyperplasia and consolidation	Pulmonary edema, hemorrhage and mucus suppository	Recruitment and infiltration of inflammatory cells		
Omicron infection with daily nasal irrigation	#1	1+1+1	1+1+1	1+1+2	3+3+4	3.33
	#2	2+1+1+1	2+2+1+0	3+2+1+1	7+5+3+2	4.25
	#3	1+1+1+0	2+1+1+1	2+1+1+0	5+3+3+1	3
	#4	2+2+1+1	1+1+1+1	3+3+2+2	6+6+4+4	5
	#5	2+2+1+1	2+2+2+1	3+3+2+1	7+7+5+3	5.5
	#6	1+1+1+0	2+1+1+1	2+2+1+1	5+4+3+2	3.5
Omicron infection without nasal irrigation	#13	3+2+2+0	4+2+2+1	4+3+3+1	11+7+7+2	6.75
	#14	4+3+2+2	4+4+3+3	2+2+1+1	10+9+6+6	7.25
	#15	3+2+2+1	1+1+1+1	3+3+3+1	7+6+6+3	5.5
	#16	3+3+3+1	3+2+2+1	3+3+3+1	9+8+8+3	7
	#17	3+2+2+1	3+2+2+1	4+2+2+2	10+6+6+4	6.5
	#18	3+2+2+2	3+2+2+2	3+2+2+2	9+6+6+6	6.75
Prototype SARS-CoV-2 infection with daily nasal irrigation	#7	3+3+2+2	4+3+2+2	4+3+3+2	11+9+7+6	8.25
	#8	3+3+2+2	4+3+2+2	4+3+2+1	11+9+6+5	7.75
	#9	3+3+2+2	4+3+3+2	4+3+3+2	11+9+8+6	8.5
	#10	3+3+2+2	3+2+2+2	3+3+2+2	9+8+6+6	7.25
	#11	3+2+2+0	3+3+2+1	3+3+2+1	9+8+6+2	6.25
	#12	4+3+2+1	4+3+3+1	3+3+3+2	11+9+8+4	8
	#19	4+4+4+3	4+4+4+4	3+3+2+2	11+11+10+9	10.25

Prototype SARS-CoV-2 infection without nasal irrigation	**#20**	4+4+4+2	3+3+3+2	4+3+3+2	11+10+10+6	9.25
	**#21**	4+4+3+1	4+4+3+2	4+4+3+1	12+12+9+4	9.25
	**#22**	3+3+2+2	3+3+2+2	4+4+3+2	10+10+7+6	8.25
	**#23**	4+3+3+2	3+3+3+2	4+4+3+3	11+10+9+7	9.25
	**#24**	2+2+2+1	2+2+2+1	3+3+2+2	7+7+6+5	6.25

Table S2. Comprehensive pathological scores of the SARS-CoV-2 Omicron variant infected hamsters sacrificed at 5 dpi, related to Figure 2.

Group	No.	Pathological lesions			Comprehensive pathological score	Average
		Alveolar septum hyperplasia and consolidation	Pulmonary edema, hemorrhage and mucus suppository	Recruitment and infiltration of inflammatory cells		
Omicron infection with nasal irrigation at 1 and 3 dpi	**#25**	2+2+1+0	2+2+1+1	2+2+1+1	6+6+3+2	4.25
	**#26**	2+2+2+1	2+2+1+1	2+2+2+1	6+6+5+3	5
	**#27**	1+1+1+1	2+2+1+1	2+2+1+1	5+5+3+3	4
	**#28**	2+2+1+0	2+2+1+1	2+2+1+1	6+6+3+2	4.25
	**#29**	3+1+1+0	3+2+1+1	3+1+1+1	9+4+3+2	4.5
	**#30**	2+1+1+1	2+2+2+1	3+1+1+1	7+4+4+3	4.5
Omicron infection without nasal irrigation	**#31**	3+3+3+1	2+2+2+2	3+3+3+2	9+8+8+5	7.5
	**#32**	3+3+2+2	3+3+3+3	4+4+3+3	10+10+8+8	9
	**#33**	3+3+2+1	2+2+1+1	3+3+2+1	8+8+5+3	6
	**#34**	3+2+1+1	2+2+1+0	3+3+1+1	8+7+3+2	5
	**#35**	3+3+1+1	2+2+1+1	3+3+2+0	7+7+4+2	5
	**#36**	2+1+1+1	1+1+1+1	1+1+1+1	4+3+3+3	3.25

Table S3. The gene-specific primers (5' to 3') used for RT-PCR for cytokines, related to Figure 4.

Genes	Forward	Reverse
**Hamster IFN-γ**	TGTTGCTCTGCCTCACTCAGG	AAGACGAGGTCCCCTCCATTC
**Hamster IL-6**	AGACAAAGCCAGAGTCATT	TCGGTATGCTAAGGCACAG
**Hamster IL-10**	GGTTGCCAAACCTTATCAGAAATG	TTCACCTGTTCCACAGCCTTG
**Hamster TNF-α**	TGAGCCATCGTGCCAATG	AGCCCGTCTGCTGGTATCAC
**Hamster ISG15**	AAAGCCTACAGCCATGACCT	TTAGTCAGGGGCACCAGGAA
**Hamster MX1**	GCGCTTCCAGACTCTTCTGA	CCTAAGATACATGCGATGGCG
**Hamster γ-actin**	ACAGAGAGAAGATGACGCAGATAATG	GCCTGA ATGGCCACGTACA
